# Recent Advances in Ultrasound Diagnosis of Carpal Tunnel Syndrome

**DOI:** 10.3390/diagnostics10080596

**Published:** 2020-08-15

**Authors:** Yuichi Yoshii, Chunfeng Zhao, Peter C. Amadio

**Affiliations:** 1 Department of Orthopaedic Surgery, Tokyo Medical University Ibaraki Medical Center, Ami, Ibaraki 300-0395, Japan; 2 Department of Orthopaedic Surgery, Mayo Clinic, Rochester, MN 55905, USA; zhaoc@mayo.edu (C.Z.); pamadio@mayo.edu (P.C.A.)

**Keywords:** carpal tunnel syndrome, ultrasound, elastography, speckle tracking

## Abstract

With the widespread use of high-resolution ultrasonography, ultrasonic examination has been shown to be useful as a diagnostic method for carpal tunnel syndrome. The main advantages of ultrasonography are that it is simple, quick, non-invasive, and economical. Another advantage is that tissue dynamics can be observed with real-time imaging. In recent reports, it has been shown that ultrasonic examination can provide similar diagnostic accuracy as nerve conduction study in the diagnosis of carpal tunnel syndrome. It has been expected that ultrasound demand in daily medical care will continue to increase. Ultrasonography in carpal tunnel syndrome shows an enlarged median nerve in proximal carpal tunnel, thickening of the flexor retinaculum, and edema around flexor tendons in cross-sectional images. In addition, with the introduction of new technologies such as ultrasonic elastography and speckle tracking, it has become possible to quantify dynamics and material property changes of nerves, tendons, and their surrounding structures. In this review, we describe recent advancements of carpal tunnel syndrome diagnosis based on ultrasound dynamic images, and discuss its pathology.

## 1. Introduction

Carpal tunnel syndrome (CTS) was first reported in 1854 by Paget as a posttraumatic entrapment neuropathy at the wrist joint [1]. In the 1960s, Phalen popularized the far more common idiopathic CTS, and increased the recognition of this syndrome [2]. At present, CTS is recognized as the most frequent peripheral neuropathy, and its prevalence is reported to range from 0.2–4% of the general population [3,4,5,6].

CTS is caused by compression of the median nerve at the wrist as the nerve passes through a narrow osteofibrous canal, along with the nine finger flexor tendons. There are many potential causes for CTS, such as malunion after distal radius fracture, rheumatic synovitis, amyloidosis, diabetes, pregnancy, or soft tissue tumor. But most of the time, CTS is idiopathic. Traditionally, the diagnosis of carpal tunnel syndrome is established by the history, clinical symptoms, and physical examination. Clinical findings include numbness and impaired sensation of the innervation area of median nerve, brachialgia paraesthetica nocturna, thenar muscle atrophy, sometimes swelling of the palmar side of the wrist, and the results of provocative tests such as Phalen’s maneuver or Tinel’s sign. Electrophysiological (EP) tests are useful when the diagnosis is unclear, there are confounding neurological disorders such as radiculopathy or polyneuropathy, or to quantify disease severity. However, the invasiveness and false-negative rate of EP tests have led to a search for other, less invasive, and more convenient diagnostic options [7,8,9,10,11,12].

Recent technological advances in ultrasonography have improved its image quality as well as affordability, leading to an increased adaptation of ultrasound evaluation of nerve entrapment syndromes. There has been an expansive growth of literature on the topic. A PubMed search in July 2020 with the keywords of “carpal tunnel syndrome” and “ultrasound” for the period from 2000 to 2020 yielded 1212 results. The objectives of this review were to provide an overview of the recent literature on the ultrasound assessment of CTS in the last two decades, to discuss their contribution to the understanding of CTS pathophysiological mechanisms, and to consider future perspectives on the use of ultrasound imaging in patients with CTS. For inclusion in this review, a PubMed search was conducted using keywords “carpal tunnel syndrome” and “ultrasonography” or “ultrasound” from 2000 to 2020. For the conventional assessment of static ultrasound (Section 2), the references of the meta-analysis and systematic review were evaluated for additional relevant articles. The articles excluded were those that did not address ultrasound diagnosis of CTS from the titles. The articles included were those that reported the accuracy of diagnosis. Since this review focused on the recent advancement in ultrasound diagnosis of CTS, extra keywords’ search of “elastography” or “dynamic assessment” or “motion assessment” and “carpal tunnel syndrome” were performed and related articles were included for the Section 3, Section 4 and Section 5.

## 2. Static Cross-Sectional Ultrasound for CTS Assessment

**Figure 1:**
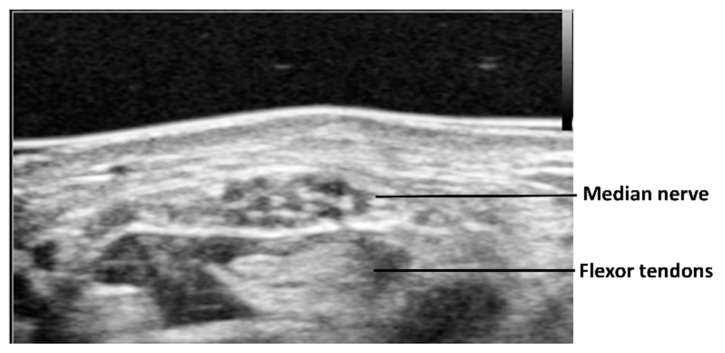
A typical ultrasound image of median nerve at proximal carpal tunnel.

In the 1990s, it was well established in the literature that static ultrasound imaging could detect pathologies such as thickening and echogenicity alteration of the flexor tendons [13] and flexor retinaculum [14], synovial proliferation, swelling of the median nerve in the proximal part of the carpal tunnel, and flattening of the median nerve in the carpal tunnel [15,16,17]. Also, restricted motion of the median nerve with different finger and wrist positions was demonstrated in patients with CTS [18,19]. Using cross-sectional ultrasound imaging, the median nerve is observed as a honeycomb structure surrounded by the hyperechoic epineurium (Figure 1). Morphological changes of the median nerve are expected in CTS, as the compression of the surrounding nonrigid structures alters its shape. This effect results in a reduction of nerve volume at the site of compression and increased size proximal (and sometimes distal) to the compression. Thus, the cross-sectional area of the median nerve is the most commonly applied criterion for diagnosing CTS on ultrasound. The cross-sectional area of the median nerve is obtained by visualizing the nerve perpendicular to its axis and tracing the nerve within its hyperechoic epineurium. It is easy to visualize the median nerve at the proximal carpal tunnel. However, at the middle or distal carpal tunnel, it is difficult to visualize. This is because the median nerve is deeper in the proximal palm than it is in the distal forearm or distal palm, and in the proximal palm it is covered by the thick flexor retinaculum. One report measured the cross-sectional area of the median nerve at three different levels: The distal edge of the flexor retinaculum, the hook of the hamate, and at the wrist crease [20]. The cross-sectional area was smaller in the central part than in the distal and proximal parts of the carpal tunnel, suggesting that the median nerve had an hourglass-like shape in cases of CTS. The median nerve is enlarged not only at the proximal to the carpal tunnel but also at the distal. Of course, this finding is frequently observed by surgeons clinically, during open carpal tunnel release surgery.

Because it is easiest to visualize, the most often reported ultrasound parameter used to diagnose CTS is the cross-sectional area of the median nerve at the proximal carpal tunnel. According to an evidence-based guideline, measurement of the median nerve cross-sectional area at the wrist was found to have Level A evidence to support a diagnosis of CTS [21]. In addition, it was suggested in this guideline that ultrasound adds value to EP testing in assessing CTS, as it can detect structural anomalies. There is a wide variation in the reported cutoff value for the cross-sectional area on ultrasound varying from 9 to 14 mm^2^ [22,23,24,25,26,27,28,29,30,31,32,33,34,35,36]. The sensitivity varied from 57–94% and specificity varied from 57–98%. One meta-analysis reported the pooled sensitivity and specificity of ultrasound for the diagnosis of CTS, were 77.6% (95% confidence interval (CI) 71.6–83.6%) and 86.8% (95% CI 78.9–94.8%), respectively [37]. The average of the diagnostic accuracy was 82.2%. Another recent meta-analysis revealed that its diagnostic odds’ ratio could be up to 31.11 (95% confidence interval, 20.42–47.40) [38]. The differences of the diagnostic accuracy and cutoff values are affected not only by disease severity but also by the nerve size related to height, sex, weight, age, race, and quality of visualization. In fact, age, sex, and race differences in nerve size have not been well investigated.

**Figure 2:**
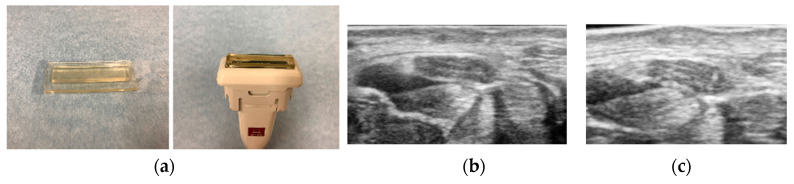
Use of acoustic coupler for the uneven skin surface. (**a**) Acoustic coupler, (**b**) an example image of median nerve without acoustic coupler, (**c**) an example image of median nerve with acoustic coupler. Fascicular pattern can be visualized with the acoustic coupler.

The visualization of the median nerve from proximal to distal carpal tunnel requires a high-quality ultrasound machine and good ultrasound technique to clearly determine the margin of the nerve. Clear visualization of the median nerve at middle or distal carpal tunnel is sometimes impaired by the inherent slight curvature of the palm, the thickness of the skin, subcutaneous fat, the relatively deep location, and the oblique course of the nerve. This may explain why previous studies have not fully assessed the usefulness of cross-sectional area distal to the wrist crease. One possible solution to improve image quality in this area is using an acoustic coupler for the transducer (Figure 2). This allows good adaptation of the transducer to the skin and can obtain a clear image of the median nerve. Another approach to quantifying focal nerve enlargement is the median nerve wrist-to-forearm cross-sectional area ratio, which allows a patient to serve as their own internal control. The diagnosis of CTS improved when using the wrist-to-forearm ratio as opposed to a diagnosis established only on the basis of a single measurement of the cross-sectional area at the level of the wrist [39]. In that study, the cross-sectional areas of the median nerve were measured at the wrist level and approximately 12 cm proximally from the wrist. The wrist-to-forearm ratio was 2.1 ± 0.5 in patients with CTS and 1.0 ± 0.1 in asymptomatic volunteers. A wrist-to-forearm ratio of 1.4 or more showed 100% sensitivity for detecting patients suffering from CTS. Also, it may be possible to compare the median nerve size with the healthy side if the case is unilateral. This may be a reasonable option to standardize the assessment of cross-sectional area of median nerve for CTS diagnosis.

Evaluations of the median nerve should not be limited around the wrist. It should be extended distal to proximal. Sometime additional or different causes of median nerve neuropathy may be detected, e.g., soft tissue tumor, pronator teres syndrome. Different anatomical variations in the carpal tunnel also have been observed with ultrasound examination. These include a persistent median artery, a bifid median nerve, variations of the motor branch and the palmar cutaneous branch of the median nerve, an anomalous muscle belly of the flexor digitorum superficialis, a reversed palmaris longus muscle, the Martin–Gruber anastomosis, and the Linburg–Comstock syndrome, to name a few [40,41]. In case of CTS associated with persistent median artery, color Doppler image may be useful. Since the strength of ultrasound is to visualize presurgical anatomy and these anatomical variations may be relevant to the surgical treatment of CTS, they should be carefully sought during ultrasound assessment.

## 3. Dynamic Ultrasound for CTS Assessment

In the carpal tunnel, the subsynovial connective tissue (SSCT) connects tendons to tendons and tendons to the median nerve. The SSCT plays an important role in reducing the sliding resistance between tendons and in maintaining blood flow to the moving tendons. It has been reported that the SSCT in patients with idiopathic CTS shows tissue proliferation such as collagen bundle degeneration, fibrosis and edema, and vascular smooth muscle thickening and thrombus formation, but without any inflammatory cells [42,43]. In addition, the SSCT of CTS patients have been shown to have reduced viscoelasticity compared to healthy subjects [44]. These changes may affect median nerve dynamics and morphology during flexor tendon motions. In recent years, the median nerve stress according to the finger and wrist motion has been attracting attention as a risk factor for the development of carpal tunnel syndrome.

**Figure 3:**
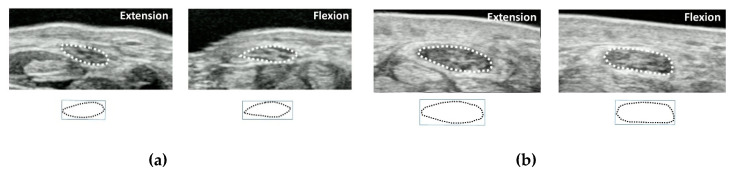
An example image of the median nerve shape in finger extension and flexion positions. (**a**) Healthy subjects, (**b**) carpal tunnel syndrome (CTS)patients. Reprinted from [48].

In order to clarify the morphological changes and displacements of the median nerve during flexor tendon sliding in CTS patients, cross-sectional dynamic analysis was introduced [45,46,47]. In this method, the transducer is placed parallel to the wrist crease at the proximal part of the carpal tunnel (Figure 3). The subject is instructed to imitate a grasping motion from finger extension to finger flexion (until the fingertip touches the palm) while recording the ultrasonic dynamic image of the tendons, median nerve, and SSCT. The final position of each extension and flexion position is the target of image evaluation, and the morphology and displacements of the median nerve are traced. The median nerve area, circumference, aspect ratio, and circularity are evaluated. Circularity is a feature that measures the complexity of a shape based on area and perimeter, and is defined as (square of median nerve circumference)/(median nerve area × 4π). Therefore, in the case of a perfect circle, the degree of circularity becomes one. Each parameter was compared between healthy subjects and CTS patients and between finger extension and flexion. For both finger extension and flexion positions, there were significant differences between healthy subjects and CTS patients in the median nerve area, circumference, and circularity. As for the deformation patterns in the healthy subjects, when the finger flexed, the aspect ratio decreased and the circularity increased. On the other hand, the aspect ratio increased and the circularity decreased in the CTS patients during finger flexion [46,47,48]. It was found that healthy subjects show morphological changes such that nerves are flattened when the fingers are flexed but become more like a circle in CTS patients.

These facts show that in a healthy person the median nerve can be morphologically deformed by tendon motion accompanying finger motion. The change in the cross-sectional area is also considered to be a movement of the median nerve in the longitudinal direction. On the other hand, in CTS patients it can be seen that the morphological changes of the median nerve associated with finger movement are small. Dynamic ultrasound can detect these changes of mobility and flexibility of median nerve in the pathological conditions of CTS.

## 4. Ultrasound Elastography for CTS Assessment

Ultrasound elastography is a technique for imaging the hardness of a tissue by an ultrasonic examination. There are two main principles for ultrasound elastography, which are categorized by the measured physical quantity, strain imaging and shear wave imaging [49,50,51]. Strain imaging is used to evaluate the degree of displacement when applying an external force to the tissues [49]. In strain imaging, tissue displacement is calculated by processing radiofrequency datasets obtained before and after compression [51]. When compression is applied to an elastic tissue, a method evaluates the degree of the deformation, utilizing the characteristic that the deformation is large when the tissue is soft and is small when the tissue is hard. In contrast to strain imaging, shear wave imaging measures physical tissue displacement parallel to the applied normal stress [49]. It employs a dynamic stress to generate shear waves in the parallel or perpendicular dimensions. Measurement of the shear wave speed results in qualitative and quantitative estimates of tissue elasticity. While these concepts are relatively straightforward to apply in a tissue of uniform physical properties, such as breast or liver [52,53], application becomes complex when properties are not uniform [54] or are affected by direction, such as occurs in the carpal tunnel, where the SSCT, tendons, and nerves all have different properties and these properties, and ultrasound wave propagation, differ in the transverse and longitudinal directions.

**Figure 4:**
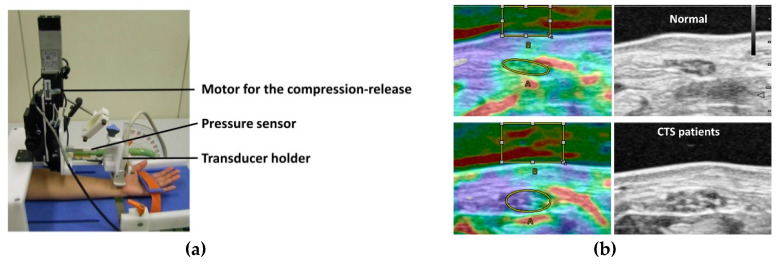
Pressure-monitor ultrasound system for the quantitative assessment of median nerve strain. (**a**) Pressure-monitor ultrasound system, (**b**) elastographic images of CTS patients and normal controls. Upper row shows the image of a normal subject. Lower row shows the image of a CTS patient.

Several ultrasound elastography studies have assessed changes of median nerve mechanical properties in CTS [55,56,57,58,59,60,61,62,63]. In a recent meta-analysis, regardless of the ultrasound elastography mode, median nerves at the wrist level were consistently stiffer in patients with CTS than those in healthy controls [64]. There were several different parameters used to evaluate material property changes of median nerve, i.e., color diagram, strain, strain ratio, shear wave velocity, and shear modulus. There are various cutoff values for each method, due in part, as well, to differences in the measurement principles with different ultrasound systems. For strain imaging, strain of the median nerve is the most generally applied parameter. In some strain elastography-based studies, mean tissue strain in the CTS patients group was significantly lower than in the healthy controls [59,63,65]. Although there were no clear descriptions for the accuracy, the calculated accuracy of the strain measurements was 56.5–70% with the cutoff values of 0.06–0.19. Another approach of using strain elastography is the measurement of color pixels of elastographic images. A higher density of blue pixels (signifying harder nerves) and a lower density of red pixels (signifying softer nerves) were found in the CTS population [55,56]. However, considering the substantial variance in the results in healthy controls, it has been proposed to diagnose CTS based on the wrist-to-forearm ratio of median nerve stiffness, rather than on the basis of the absolute stiffness of the nerve, similar to the cross-sectional area measurements [39]. Since the applied force affects the results of strain measurements, some studies used strain ratio, which is a parameter measuring the ratio of median nerve strain and comparative structures, i.e., fat, tendon, or acoustic coupler [57,65,66,67]. In another study, to standardize the strain measurement, special equipment was developed [58] and subsequently modified with a pressure-monitor ultrasound system [68] (Figure 4). With this equipment, the applied force and displacement of the transducer were more reproducible, and strain with applied force can be evaluated quantitatively.

**Figure 5:**
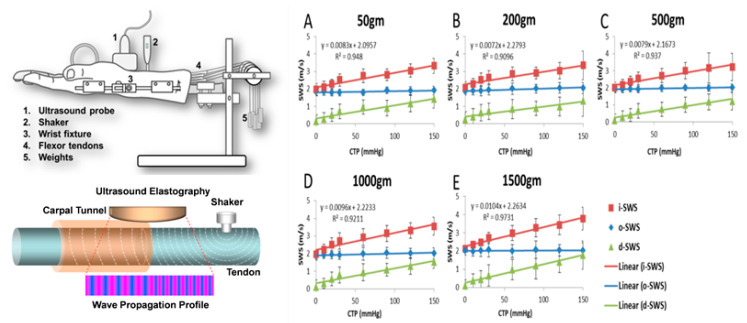
Shear wave elastography for the estimation of carpal tunnel pressure. Left top and bottom figures show the experimental setting and ultrasound elastography measure of third digit FDS tendon. (**A**–**E**) demonstrate the wave speed of the tendon within and outside carpal tunnel region. Reprinted from [70].

In studies of shear wave elastography, shear wave velocity was significantly lower [61] and shear modulus was significantly higher [60,62,69] in CTS patients compared to healthy controls. When using shear wave elastography, the longitudinal image is sensitive to changes in median nerve elasticity. Shear wave elastography evaluates tissue stiffness by detecting the propagation speed of shear waves, which is related to the stiffness of the medium. In a study with quantitative data, shear modulus of median nerve was reported as 66.7 kPa in CTS patients and 32.0 kPa in control subjects, with a cutoff value of 40.4 kPa having an accuracy rate of 91.7% [60]. In addition, it has been shown that shear wave elastography has a potential to estimate the carpal tunnel pressure [70,71] by evaluating tendon shear wave speeds inside and outside the carpal tunnel in a cadaveric model. The results showed that the shear wave speed inside carpal tunnel increases linearly with increased carpal tunnel pressure (Figure 5). There are several limitations for strain imaging and shear wave imaging. For both techniques, the penetration and reverberations are limited because they are affected by push-beam quality and the reliability of displacement estimates. It is difficult to evaluate tissues covered with hard structures. It has been suggested that the measurements produced by different systems must not be compared in clinical practice to monitor a patient and the threshold values must only be used in an analysis carried out with the same system and same transducer [72]. For the strain imaging, the applied force affects the results of strain measurements. Therefore, the operator needs careful attention to apply constant pressure to the tissue. For shear wave imaging, which detects the propagation speed of shear waves, the measurement of deeper structures may not be accurate.

It is known that chronic nerve compression results in intra-neural edema, followed by peri-neural thickening and nerve fiber changes with Wallerian degeneration [43,73]. Clinically, nerve swelling is often observed, but ultrasound elastography has made it possible to quantitatively show changes of tissue elasticity. The hardness information provided by ultrasound elastography is considered to be an index for clinically reflecting histological changes in the median nerve and surrounding structures.

## 5. Speckle Tracking for CTS Assessment

**Figure 6:**
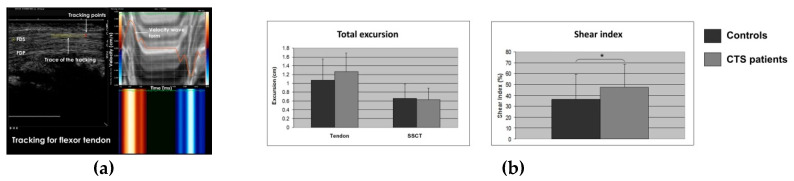
Speckle tracking ultrasound for the assessment of CTS. (**a**) An example image of speckle tracking, (**b**) tendon and subsynovial connective tissue excursions, and shear index. Reprinted from [79,80].

Another approach for the ultrasound assessment of CTS uses speckle tracking. Speckle tracking is an application of pattern matching technology to ultrasonic images. A reference image for dynamic analysis is set in the initial frame and then the region in the next frame where the speckle pattern is most similar to the reference image is estimated, and the moving speed and direction of the tissue are thus quantitatively evaluated, frame by frame. By reconstructing the deformation and motion of the speckles, the motion of fluid and tissues can be analyzed. The accuracy of speckle tracking and its clinical utility for the assessment of cardiac function has been demonstrated in cardiac imaging studies [74,75,76,77,78]. This speckle tracking method was applied to quantitative evaluation of the shear stress of flexor tendon and surrounding SSCT during finger motion in the carpal tunnel [79,80] (Figure 6). In healthy controls and CTS patients, the transducer was placed parallel to the gliding direction of the flexor tendon of the finger at the proximal part of the carpal tunnel, and the long-axis image of the flexor tendon was displayed. Images were recorded when the fingers were flexed from the extended position and extended from the flexed position. Finger extension-flexion-extension was set as one cycle, and tracking points were set on the flexor tendon and SSCT, which located on the margin of flexor tendon. The movement distance in each major axis direction was measured. Shear index was defined as {(moving distance of tendon-moving distance of subsynovial connective tissue)/moving distance of tendon} × 100 (%) and compared between healthy subjects and CTS patients. In this study, the shear index was significantly higher in CTS patients (36.3%/47.8% in healthy subjects/CTS patients, respectively) [80]. This suggests that the shear stress of tendon and SSCT is increased in CTS patients. Since the SSCT of CTS patients has been shown to have reduced viscoelasticity compared to healthy subjects, speckle tracking measurement of SSCT, tendon, and nerve shear index may be a way to non-invasively assess the amount of fibrosis within the carpal tunnel and to correlate this physiological property with the outcomes of various treatments, such as stretching exercises or steroid injections.

Speckle tracking has the advantage over Doppler imaging that tissue dynamics can be analyzed regardless of the angle between the object and the ultrasound transducer [79]. Speckle tracking has the potential to show the difference in shear stress of the tendon and its surrounding tissue between CTS patients and healthy subjects. However, tracking a flexor tendon that moves in three dimensions requires the accurate placement of the transducer in the gliding direction of the tendon. There are still issues to be considered for clinical application of speckle tracking analysis for CTS, which may not be resolved until high-resolution, real-time, 3D dynamic imaging (really, 4D imaging) is available as a clinical option.

## 6. Future Perspectives

As described so far, recent advancements in the ultrasound evaluation of patients with CTS provide more pathophysiological information about the median nerve and surrounding structures. This information improves the diagnostic accuracy and deepens our understanding of the pathology of CTS. But there are still some challenges in the ultrasound assessment. First, since the ultrasound methods are known to have operator dependency, development of standardized protocols for image analysis is needed. Second, it is important to clarify morphological variations in the median nerve depending on race, sex, and physique. It is also needed to investigate diagnostic significance of ultrasound in different disease populations, like diabetes and chronic renal failure patients. Since there are reports that showed differences in the carpal tunnel characteristics [81,82], CTS patients related with these diseases may need to set different cutoff values. The cutoff value for the measurement due to these differences should be determined. Third, the measurement parameters for the longitudinal assessment should be investigated. The longitudinal images are useful in case of focal compression of the nerve because they show the compressed portion of the nerve as well as the proximal and/or distal swollen portion [41]. However, it is rarely used as a quantitative parameter. This is because there is no clear landmark that indicates the positional relationship with the wrist surface and it is relatively difficult to visualize long-axis view of median nerve. Fourth, it is still unclear how these ultrasound findings correlate with disease progression. It can be considered that these findings depict pathological anatomy and kinematics associated with CTS. It remains to be seen, though, whether it is possible to predict outcome or identify risk factors based on ultrasound findings. Fifth, the role of ultrasound exam in the decision for treatment options is still unclear. Ultrasonography is useful as an initial diagnostic test to detect median nerve size, anatomical abnormalities, and space-occupying lesions. Since the ultrasound evaluations do not directly reflect the neurological functional impairment, physicians will still need EP and other clinical studies to complement the ultrasound findings. Until a single diagnostic method is able to accurately predict treatment outcomes and assess prognosis, a comprehensive diagnostic investigation will still be needed. Lastly, postoperative findings should be more focused in relation to the therapeutic effects. Some studies confirmed reduction of median nerve size and recovery of material properties [83,84,85]. However, it is relatively few reports. Prognostic potential of ultrasound should be evaluated more intensively. Research related to these ultrasonic examination methods and treatment results will be useful in future studies on the prevention and prognosis of CTS. Despite the large amount of information that accompanies the improvement of ultrasound technologies, these issues remain unclear. In order to solve these problems, we would like to suggest conducting a large-scale epidemiological survey that evaluates the clinical findings and ultrasound assessment of CTS.

## 7. Conclusions

Recent advancements in the ultrasound assessment of CTS have been reviewed. It is well accepted currently that static ultrasound measurement of median nerve cross-sectional area is helpful in the diagnostic evaluation of CTS patients as the initial diagnostic test and may, in some cases, at least, eliminate the need for EP testing. In addition, ultrasound imaging, especially dynamic imaging, has made it possible to investigate CTS pathophysiology. That is, the morphological change pattern of the median nerve due to finger movement is different in CTS patients from that of healthy persons. In addition, the elastic modulus of the median nerve is decreased in CTS patients, and the shear stress of the SSCT relative to adjacent tendons is increased in CTS patients. At present, these analyses require work after image acquisition, but it is expected that each parameter will be measurable more automatically using software on the ultrasound equipment in the future. It is expected that various causes of CTS will be clarified based on these evaluation criteria and that ultrasound measures will be applied to treatment selection and outcome assessment in patients with CTS in the future.
